# Dermatophytosis in companion animals: A review

**DOI:** 10.14202/vetworld.2020.1174-1181

**Published:** 2020-06-22

**Authors:** Alsi Dara Paryuni, Soedarmanto Indarjulianto, Sitarina Widyarini

**Affiliations:** 1 Department of Pathology, Faculty of Veterinary Medicine, Universitas Gadjah Mada, Yogyakarta 55281, Indonesia; 2 Department of Internal Medicine, Faculty of Veterinary Medicine, Universitas Gadjah Mada, Yogyakarta 55281, Indonesia; 3 Department of Pathology, Faculty of Veterinary Medicine, Universitas Gadjah Mada, Yogyakarta 55281, Indonesia

**Keywords:** clinical signs, cortisol, cytokine, dermatophytosis, immune response, pathogenesis

## Abstract

Dermatophytosis, a zoonotic disease, is caused by fungi of three main genera, namely, *Micropsorum, Trichophyton*, and *Epidermophyton*. Specific lesions of dermatophyte infections are localized in the face, legs, and/or tail. Skin lesions in infected animals demonstrate localized alopecia, erythema, and crust, which are more commonly known as ringworm. Factors that affect dermatophytosis include the dermatophyte species; virulence factors of the agent; and the immune status, age, and sex of the host. High levels of cortisol and pro-inflammatory cytokines have also been reported to play an important role in dermatophyte infection. This review aims to explore and understand factors that affect dermatophyte infection with an emphasis on the prevalence, clinical signs, pathogenesis, immune response, and the roles of cortisol and cytokines in companion animals infected by a dermatophyte.

## Introduction

Dermatophytosis is a zoonotic disease caused by fungal infection of a species of dermatophyte [1,2]. Dermatophytosis is more commonly known as ringworm, which is macroscopically characterized by multifocal alopecia and crust on the skin with a specific formation [3,4]. This disease is distributed globally and has gained special attention in public health [2,5]. Dermatophyte infections in humans occur after contact with contaminated products or specimens, such as soil, hair, or crust on the epidermal layer of infected animals [5].

Annual cases of dermatophytosis have increased not only in humans but also in animals, particularly dogs and cats [6]. In Indonesia, cases of dermatophytosis are reported to be more prevalent in adult female cats and kittens than male cats [1]. The results of the research on dogs showed that 34% of dogs in Yogyakarta, Indonesia, were positive for dermatophytosis [7]. In Europe, the incidence of dermatophytosis in dogs and cats ranges from 20 to 30% [8]. The results of various studies concluded that the main species causing dermatophytosis in pets is *Microsporum canis*, in 81.8%-97% of the cases [1,2,6]. The incidence of zoonotic dermatophytes *M. canis* was the highest in dogs, cats, and human (60.0%) compared to other species [9]. High levels of cortisol over a long period of time modulate the immune response and induce immune suppression as the result of lymphatic tissue atrophy [8]. Stress also induces cortisol production and results in inhibition of the Th1 cytokine pro-inflammatory response and upregulates the Th2 anti-inflammatory cytokine response [8,10]. It has been reported that high levels of cortisol in cats, as a consequence of stress, alter host immune responses and might promote the dermatophyte infections [10].

This review discusses the prevalence, clinical signs, pathogenesis, immune responses, and roles of cortisol and cytokines in companion animals infected with a dermatophyte.

### Etiology and Prevalence

**Figure-1:**
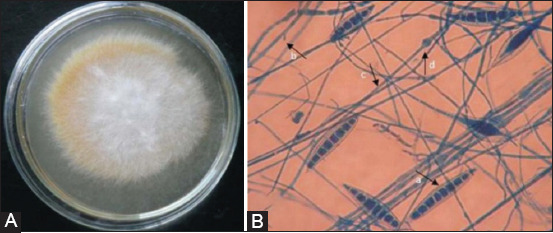
Colony of *Microsporum canis* on SDA media (14 days). Colony is cottonny, white to buff in color; with increasing age becomes brownish yellow (A); artrospora of *M. canis* viewed under microscope (B): a. macroconidia, b. microconidia, c. hyphae, d. chlamydoconidia, viewed under microscope with lacto phenol cotton blue staining [7].

**Figure-2:**
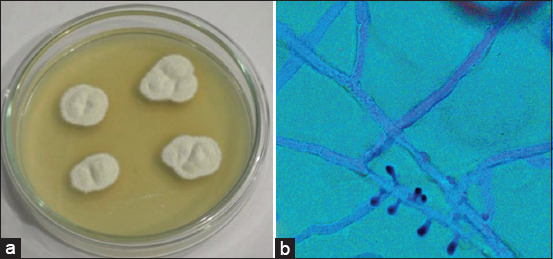
Colony of *Trichophyton rubrum* on SDA media (14 days). Colony is white, fluffy, with a central umbo and flat periphery (a); microaleurispores of *T. rubrum* are scanty and slim viewed under microscope with lacto phenol cotton blue staining (b) (Private Documentation, 2020).

Dermatophytes consist of 40 species of fungi derived from three genera, namely, *Micropsorum, Trichophyton*, and *Epidermophyton* [11,12]. The two genera of fungi which are the main causes of dermatophytosis in animals (especially in dogs and cats) are *Microsporum* spp. and *Trichophyton* spp. *M. canis, Microsporum gypseum, Trichophyton mentagrophytes, Trichophyton equinum, Trichophyton verrucosum*, and *Microsporum nanum* species of fungi have important roles in veterinary medicine [3,13,14]. Common dermatophytes that infect small animals include *M. canis, T. verrucosum*, and *T. mentagrophytes* [15]. This study also showed that *T. mentagrophytes* is often found in guinea pigs and rabbits [15]. A colony of *M. canis* and *T. mentagrophytes* is shown in Figures-1 and 2, respectively.

More than 90% of dermatophyte infections in cats are caused by *M. canis*. *M. canis* also infects humans and other animal species such as dogs, cattle, horses, pigs, goats, rabbits, guinea pigs, apes, monkey, tigers, and mice [6]. A study in Lisbon, Portugal, showed that 82% of 89 cats positive for dermatophytosis were infected with *M. canis* [16]. Subclinical infection of *M. canis* also occurs in long-haired juvenile cats [6]. An Indonesian study reported that 17 (56.7%) of 30 cats with dermatitis infections are positive for dermatophytosis caused by *M. canis* [1]. Several studies also showed that dermatophytosis occurs more often in female animals than in males [3,17]. Thus, sex and age play important roles in the prevalence of dermatophytosis in animals.

In a retrospective study in dogs (1970-2002), *T. mentagrophytes* were found in 66 cases (66/3854, 1.7%), while *M. canis* was diagnosed in 840 cases (840/3854, 21.8%) [18]. A study to determine dermatophyte distribution in dogs and cats in West Turkey found that 14.4% of samples tested were positive for dermatophytosis in a sample size of 326 [19]. In Italy, the reported prevalence of dermatophytosis was 7.5%-20.5% in dogs and 24.7-33.3% in cats [8]. In Eastern India, out of 1209 samples from dogs and 292 samples from cats, 253 (20.93%) and 109 (37.33%), respectively, were positive for dermatophyte spores [20]. It was reported that 34% dogs in Yogyakarta, Indonesia, are positive for dermatophytosis [7]. A study in Baku in the Middle East showed that 108 of 193 dogs and cats were infected with a dermatophyte [21].

Dermatophytosis occurs not only in small animals but also in other species of animal. A study of dermatophytosis in farm animals done in Beheira, Egypt, demonstrated that 74% of 150 samples collected from different species of animals were ­dermatophyte positive. The highest dermatophytosis case rates reported occurred in sheep (78%), followed by buffaloes (76%), cattle (72%), and horses (68%) [3]. The samples from female camels farmed in Central Saudi Arabia showed a dermatophytosis prevalence of 11.5%. Interestingly, the higher incidence occurred at a young age [17]. A study showed that prevalence of Arabian horse dermatophytosis in Egypt was 16.8% of the total prevalence of 81% [22]. It was reported that the prevalence of dermatophytosis in cattle farms in Irbid, Jordan, ranges from 10 to 100% [23]. That study also showed that 115 (30.6%) of 375 cattle were identified as having a typical macroscopic lesion of dermatophytosis.

### Clinical Signs

**Figure-3:**
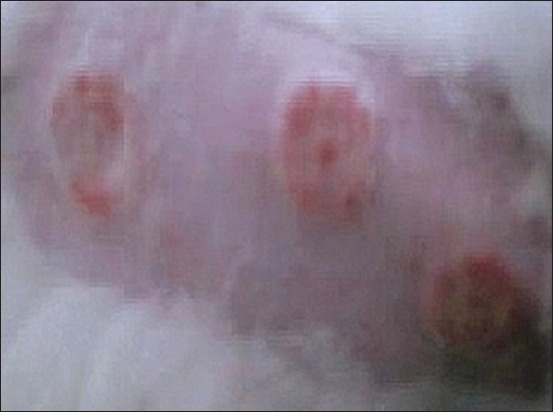
Lesions from *Microsporum canis* in body part of cat. Formation of round shape lesions in form alopecia, and erythema in the skin [7].

**Figure-4:**
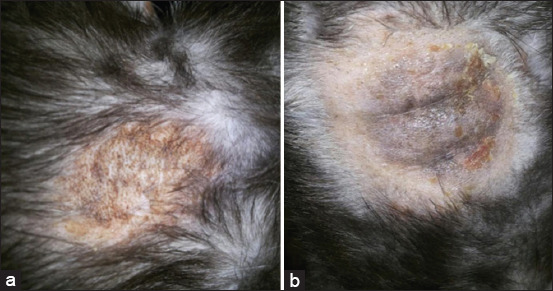
Lesions from dermatophyte infection in the body part of cat with crust and alopecia in the skin (a), hyperpigmentation (b) (Private Documentation, 2020).

Lesions in cases of dermatophytosis are variable for each species of animal [1,24-26], the most common clinical symptoms being hair loss, skin crust, erythema, and pruritus (Figures-3 and 4). Other studies showed that dermatophyte infection in dogs cause lesions localized to the face, legs, and/or tail [27]. The previous studies [1,19,26] have demonstrated that infected dogs clinically showed lesions in the skin such as multifocal alopecia, erythema, papule, pustule, scale, and crust with a distinctive formation known as ringworm. Infection of *M. canis* results in alopecia (1-3 mm) in the infected area and then permanent alopecia occurs, especially if the inflammatory reaction lasts a long period of time [28]. Lesions caused by dermatophytes can be mild to severe depending on several factors, including the infecting dermatophyte species, virulence factors, area of infection, secondary infections, and environmental conditions [29].

In rabbits, macroscopic lesions caused by fungal infection include yellowish-white dry crust formation in the face, eyelids, ears, nails, legs, and back area, alopecia, and erythema [30]. Round shaped, white, gray, or blackish brown lesions (hyperpigmentation) along with hyperkeratosis, scales, and crust are found on the skin of Balinese cattle (*Bos javanicus*) infected by dermatophytes [31].

Based on the previous studies, it can be concluded that the clinical symptoms of dermatophyte infection vary depending on infected the dermatophyte species, infected area of the body, and the species of the host**.** In general, dermatophyte infections cause the formation of lesions; namely, alopecia, crust, papula, pustule, and erythema in the skin and are usually in a round shape known as ringworm.

### Pathogenesis

At the start of infection, dermatophytes attach to the keratin tissue in the nails or hair of the host [32,33]. Virulence factors of the dermatophyte, the immunological status, and the age of the host play an important role in the progression of this disease. The young and the old among animals are reported to be more susceptible to direct or indirect dermatophyte infection [2,14].

Predisposing factors affecting dermatophytosis include the number of infective spores, frequency of transmission, health conditions, and the physiological stress experienced by animals [26,34]. The route of infection occurs through skin wounds, scars, or burns [34]. This infection occurs either directly through contact with sick cats or indirectly through blankets, bed covers, toys, cages, clothes, and other objects contaminated with spores [26,34].

The incubation period of *M. canis* ranges from 1 to 4 weeks [26]. The attachment of arthroconidia to keratin occurs maximally at 6 h after infection [34]. The rapidity of attachment of arthroconidia to keratin is affected by the number of infecting arthroconidia [35]. Germination and invasion of the stratum corneum take approximately 4-6 h [34,35,36]. The colonization processes result in various immune system reactions in the host including inflammation [37]. Subsequently, an inflammation reaction can be seen in infected skin area as redness, swelling, and alopecia [36,38].

**Figure-5:**
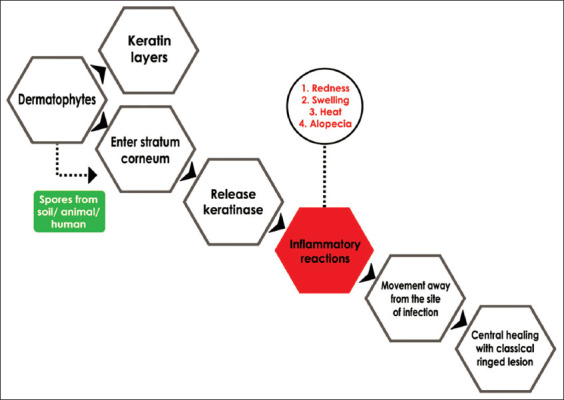
The schematic route of entry of dermatophytes into the host [38].

Dermatophytes have the ability to produce proteolytic and keratolytic enzymes that enable keratin to be used as the sole source of nutrition after colonization, facilitating fungal growth in the stratum corneum, and resulting in keratinization in the epidermis [6,33,34]. Proteolytic activity of dermatophyte by releasing serine proteinase (urokinase and activator plasminogen tissue), which causes damage of external protein of the host and facilitated by the process of injury in skin [34,38]. The ability of dermatophytes to secrete enzymes as mentioned above is one of the virulence factors of dermatophyte infection [33,36]. Hence, the ability of dermatophytes to infect the host depends on the dermatophyte species, the number of infection spores, virulence factors, and the immune status of the host. The route of dermatophyte infection is shown in Figure-5.

### Host Immune Responses to Dermatophyte Infection

Immune responses from fungal infection vary dependent on the type of infectious agent [32]. During the infection process, dermatophyte interferes the host’s defense mechanism which affects the manifestation and severity of dermatophyte infection, depends on patient’s immune response [36,39,40]. Dermatophytes have non-specific antigens, namely, glycopeptide and keratinase [33]. The glycopeptide stimulates cellular immune response, while the polysaccharide part of the glycopeptide stimulates humoral immune response [33,34].

The previous studies reported that several cell wall components of dermatophyte might act as pathogen-associated molecular patterns that are recognized by pattern recognition receptors (PRRs), including toll-like receptors (TLRs), which expressed by phagocytes and dendritic cells (DCs) [36,41-43]. Following dermatophyte infection, TLR2 signaling induces the production of inflammatory cytokines and leukotrienes, such as tumor-necrosis factor (TNF), Interleukin (IL)-1b, and IL-10 [43-48]. During the inflammatory process, PRRs activate neutrophils and macrophages to produce powerful antifungal defectins. This antifungal acts by disturbing osmotic imbalance in the fungal cells. Activated macrophages and neutrophils also release massive production of cytokines, namely, IL-1, IL-12, and TNF, which are toxic to fungal cells [40,42].

Skin is a physical barrier that prevents fungal infection through epidermal cell proliferation and keratinization. Physical barriers of the skin play an important role in immune responses at the first stage of infection by preventing and eliminating fungal infiltration on the stratum corneum [35,48,49]. During inflammatory processes in the skin, keratinocytes express the major histocompatibility complex II, cytokines, and colony-stimulating factor [36,38,47]. Other components of the immune response in the skin are Langerhans cells (LCs), DCs, macrophages, natural killer (NK) cells, and memory T cells [47]. Thus, recognition dermatophytes antigens by PRRs on phagocytes and keratinocytes result in release inflammatory cytokines, which play an important role on host immune response.

### Role of Cortisol and Dermatophyte Infection

Stress hormones (glucocorticoid, catecholamine, and neuroendocrine) can modulate various aspects of the immune system. Stress hormones also have direct effects on all cells of the immune system and influence the expression of various cytokines [50,51]. Glucocorticoid qualitatively and quantitatively induces the suppression of immune system of the host against invasive fungal infection [52]. Following stress, the concentration of glucocorticoid increases through the regulation of cellular and humoral immunity and results in host susceptibility to the fungal infection [53]. Chronic stress increases the concentration of glucocorticoid over a long period of time and downregulates the synthesis of pro-inflammatory cytokines, namely, TNF-α, Interferon (IFN)-γ, and IL-2 in animals and humans [53]. Glucocorticoids suppress the production of IL-12 by antigen presenting cells (APC) and downregulates the expression of IL-12 receptors on NK and T cells [50,51]. During fungal infection, the high concentrations of cortisol affect the normal function of the immune system [52,54]. Chronic high levels of cortisol in cats, as a consequence of unidentified stressors or for endogenous reasons, could alter immune responses and might promote dermatophyte infections in the presence of a contaminated environment [8]. A stress study in fish demonstrated the same phenomena. High concentrations of cortisol distract the fish immune responses against pathogenic agents [55]. Hence, cortisol plays an important role during fungal infection through its ability to suppress the host immune system by downregulating TNF-α, IFN-γ, and IL-2 cytokines.

### Role of Cytokines and Dermatophyte Infection

Cytokines have important effects on the activity of many cells. Cytokines are reported to be important because of their significance in regulating the immune system [56-58]. Fungal infection in skin induces Th1 cells as a humoral immune response. During fungal infection, Th1 cells secrete pro-inflammatory cytokines such as IL-2, IL-12, IL-18, and IFN-γ which stimulates phagocytic activity, cytotoxic CD4+ T cell generation, and prevents the hypersensitivity reaction [3,59,60].

CD4+ is an important component of cellular immune responses [61]. The presence of pathogenic agents will activate naïve CD4+ cells to differentiate into various Th cell groups (Th1, Th2, and Th17) [61,62]. Cytokines such as IL-1, IL-6, IL-8, IL-10, IL-15, TNF-α , and transforming growth factor-β also have immunological roles in responses to fungal infections [63]. During fungal infection, IL-12 and IL-10 act as regulators of Th cell growth and humoral immune responses [6,59,63].

Fungal infection is capable of inducing IL-17 production as a humoral immune response during the initial phase of infection [64]. IL-17 is known as a pro-inflammatory cytokine which provides complex connections between humoral and cellular immunity; this connection is the center of inflammatory responses to fungal infection [64]. IL-17 is also reported to be responsible for preventing uncontrolled fungal growth and inflammatory reactions in *M. canis* infection [65]. Studies have demonstrated that patients deficient in IL-17 are susceptible to dermatophyte infection [65-67].

IL-1 is a pro-inflammatory cytokine that is produced in response of host humoral immunity to fungal infection [68]. IL-1 consists of 11 components, including IL-1α, IL-1β, and IL-1Ra which are important for chronic inflammatory infection [69]. It was reported that the process of attachment to and fungal germination in host cell walls strongly induced the release of IL-1 [70]. IL-1 is reported to have an important role in the pathogenesis of chronic cavitary pulmonary aspergillosis (CCPA), due to its ability to induce activation of IL-1α and IL-1β pro-inflammatory cytokines [71].

IL-2 is produced from Th1 cells, CD4+, CD8+, cytotoxic T cells (CTL), DC, NK cells, and NKT cells [6,72]. IL-2 has dual functions as both an immune suppressor and/or activator system [73]. Therefore, the pro-inflammatory function from IL-2 can be inhibited by antigen-specific T *reg*. Low amounts of IL-2 and IFN-γ secretion in response to antigen produced from pathogenic fungal indicated a deficiency of cell-mediated immunity in infected patients. Low concentrations of IL-2, IL-17, IFN-γ, and IL-10 also indicated that fungal antigens were unable to induce cytokine secretion from Th1 cells, and leads to upregulated cytokine secretion from Th2 cells [72,74]. Therefore, in fungal infection low concentrations of IL-2 correlate with upregulated levels of Th2 cytokine secretion.

IL-10 has the ability to modulate natural immune responses and prevents pro-inflammatory cytokine production from Th1 and Th2 cell secretion, such as IL-2 and IFN-γ [74,75]. IL-10 is produced by monocytes and macrophages, while other cells also produce cytokines such as DC cells, B cells, CTL, γδ-T cells, NK cells, mast cells, and neutrophil granulocytes [75,76]. Several studies showed that IL-10 prevents APC (monocytes, macrophages, and DC) actions [77,78]. IL-10 is known to have a strong inhibitory effect on fungal infection. It is known that mice deficient in IL-10 more susceptible to fungal infection [79]. Patients with a fungal infection and sarcoidosis have low levels of IL-10 within the serum resulting from granuloma formation which blocks IL-10 secretion [80]. Thus, a high level of IL-10 in a host’s serum may inhibit the development of fungal infections.

IL-12 is cytokine with a role in regulating T cell response to infection. IL-12 is secreted by monocytes/macrophages, neutrophils, and also other cells [81]. IL-12 is needed for the growth of Th1 cells. The main function of IL-12 is to induce IFN- γ production from NK cells and T cells. Another function is to increase cytotoxicity of NK cells, CTL, and to differentiate *naïve* T cells into Th1-cells [81,82]. IL-12 has an important role in regulating T cell responses, initiating immune cells to differentiate into Th1, Th17, and T*reg* cells [83], and maintaining acquired immunity to microorganism infections [84]. It has been reported that IL-12 plays an important role in humoral and adaptive immune response to fungal infection. Mice with an IL-12p40 deficiency experienced systemic infection after oral infection with fungus; this study also showed that IL-12 is important in preventing the spread of the fungal infection within the host body [85]. Consequently, the high level of IL-12 in the host serum might be crucial for inhibiting the dissemination of the fungal/dermatophyte infection.

## Conclusion

This review highlights the important factors that affect dermatophyte infection in companion animals. Susceptibility to dermatophyte infection is dependent on animal age, sex, and species. The host immune system and virulence factor from the agent are also crucial for disease progression. Virulence factors from dermatophytes, namely, glycopeptide and keratinase, are responsible for initiating the host immune response. However, released stress hormone during dermatophyte infection has adverse effects on the host immune response. Stress hormone impairs the host immune response by downregulating or upregulating pro-inflammatory cytokines

## Authors’ Contributions

SW and SI conceptualized the review, drafted the review, prepared, and edited the manuscript according to the title. SW and ADP collected the literature, edited the manuscript, and finalized the manuscript. All authors read and approved the final manuscript.
